# The Latest Findings of PD-1/PD-L1 Inhibitor Application in Gynecologic Cancers

**DOI:** 10.3390/ijms21145034

**Published:** 2020-07-16

**Authors:** Omid Kooshkaki, Afshin Derakhshani, Hossein Safarpour, Souzan Najafi, Parviz Vahedi, Oronzo Brunetti, Mitra Torabi, Parisa Lotfinejad, Angelo Virgilio Paradiso, Vito Racanelli, Nicola Silvestris, Behzad Baradaran

**Affiliations:** 1 Student Research Committee, Birjand University of Medical Sciences, Birjand 9717853577, Iran; omidkoshki@gmail.com; 2 Department of Immunology, Birjand University of Medical Sciences, Birjand 9717853577, Iran; 3 Immunology Research Center, Tabriz University of Medical Sciences, Tabriz 5165665811, Iran; afshin.derakhshani94@gmail.com (A.D.); najafis@tbzmed.ac.ir (S.N.); p.lotfinezhad@gmail.com (P.L.); 4 Cellular & Molecular Research Center, Birjand University of Medical Sciences, Birjand 9717853577, Iran; safarpour701@yahoo.com; 5 Department of Anatomical Sciences, Maragheh University of Medical Sciences, Maragheh 5165665931, Iran; pa.vahedi48@gmail.com; 6 Medical Oncology Unit, IRCCS Istituto Tumori “Giovanni Paolo II” of Bari, 70124 Bari, Italy; dr.oronzo.brunetti@tiscali.it; 7 Student Research Committee, Tabriz University of Medical Sciences, Tabriz 5165665811, Iran; mitra.tbm@gmail.com; 8 Department of Immunology, Faculty of Medicine, Tabriz University of Medical Sciences, Tabriz 5166614766, Iran; 9 Institutional BioBank, Experimental Oncology and Biobank Management Unit, IRCCS Istituto Tumori Giovanni Paolo II, 70124 Bari, Italy; a.paradiso@oncologico.bari.it; 10 Department of Biomedical Sciences and Human Oncology, University of Bari “Aldo Moro”, 70121 Bari, Italy; vito.racanelli1@uniba.it

**Keywords:** immune checkpoint inhibitors, PD-1 inhibitors, gynecologic cancers, PD-L1 inhibitors

## Abstract

Gynecologic cancers account for approximately 11% of the newly diagnosed cancers in women in the United States and for 18% globally. The presence of tumor-infiltrating lymphocytes (TILs) influences the clinical outcome of cancer patients and immune checkpoint inhibitors (ICIs), including anti programmed cell death protein-1 (anti-PD-1), anti-programmed death-ligand 1 (anti-PD-L1), and anticytotoxic T-lymphocyte antigen 4 (anti-CTLA-4), which have been approved for treating different types of malignancies. Antibodies targeting the PD-1/PD-L1 checkpoint have shown dynamic and durable tumor regressions, suggesting a rebalancing of the host–tumor interaction. There are several the US food and drug administration (FDA)-approved ICIs targeting PD-1, including pembrolizumab and nivolumab, as well as those targeting PD-L1, including avelumab, atezolizumab, and durvalumab for melanoma, renal cell cancer, colorectal cancer, head and neck cancer, cervix cancer, urothelial cancer, and lung cancer. Current pre-clinical and clinical studies assessing PD-1/PD-L1 inhibitors in several gynecologic cancers have reported significant antitumor activity. In this review, we investigate pre-clinical and clinical studies that describe the safety and efficacy of anti-PD-1/PD-L1 antibodies, with a particular focus on ongoing clinical trials, analyzing the oncological outcome and adverse effects of ICIs in gynecologic cancers.

## 1. Introduction

Gynecologic cancers affect the female reproductive organs, including the cervix, ovaries, uterus, vagina, and vulva. They account for approximately 11% of the newly diagnosed cancers in women in the United States and 18% globally [1]. The most common gynecologic malignancies occur in the uterus and endometrium (53%), ovaries (25%), and cervix (14%) [2]. According to official reports, 607,000 women died in 2018 gynecologic malignancies in 2018 [3]. Surgery, radiotherapy, chemotherapy, targeted therapies, or their combination, are the main treatment choices. However, current treatments are often ineffective in advanced disease [4]. Recently, immune-targeted therapies have shown long-lasting responses in gynecologic cancers with limited treatment options and low overall prognosis [5]. There is an overwhelming amount of evidence supporting the role of the immune system in the development and growth of tumor cells. Consequently, various immunotherapeutic approaches, including vaccines, cytokines, immunomodulators, adoptive transfer of endogenous or genetically modified T cells, and immune checkpoint inhibitors (ICIs), have been assessed in the treatment of several cancers [6]. ICIs, including anti programmed cell death protein-1 (anti-PD-1)/anti-programmed death-ligand 1 (anti-PD-L1) antibodies, have attracted the greatest interest in clinical trials given the molecular basis of gynecologic cancers. There are several drug administration (FDA)-approved ICIs targeting PD-1, including pembrolizumab and nivolumab, as well as those targeting PD-L1, including avelumab, atezolizumab, and durvalumab for melanoma, renal cell cancer, colorectal cancer [7], head and neck cancer [8], cervical cancer [9], urothelial cancer [10], and lung cancer [11]. In this review, we have investigated pre-clinical studies that described the safety, efficacy, and adverse effects of anti-PD-1/PD-L1 antibodies in gynecologic cancers and the ongoing clinical trials with these agents.

## 2. PD-1/PDL1 Pathway

Malignant cells produce several antigens that stimulate an immune response, resulting in activation of cytotoxic CD8+ T cells, which then move to the tumor microenvironment [12]. There is mounting evidence that has confirmed the important role of the tumor immune microenvironment in tumorigenesis [13]. Tumor microenvironment cellular constituents include tumor-infiltrating lymphocytes (TILs), natural killer (NK) cells, macrophages, dendritic cells (DCs), and myeloid lineage cells [14]. The involvement of TILs is associated with the clinical outcome of patients with different cancers. Major components of the TILs are CD8+ and CD4+ T cells, which are the main types of TILs that can lead to effective tumor elimination [15]. Activated CD8+ T cells secrete large amounts of inflammatory cytokines, such as tumor necrosis factor (TNF)-α and IL-6, and present a high cytotoxicity against tumor cells. CD4+ T cells also secrete many cytokines that promote the differentiation of B cells into antibody-producing plasma cells [16]. High levels of CD4+ T cells and CD8+ T cells have been shown to be associated with enhanced disease-free and overall survival of cancer patients [17]. T cells also determine the ultimate amplitude and quality of the response to tumor cells through antigen recognition by the T cell receptor (TCR), which is regulated by a balance between co-stimulatory and inhibitory signals (immune checkpoints) [18]. Cytotoxic T-lymphocyte antigen 4 (CTLA-4) and programmed cell death protein-1 (PD-1) are two major immune checkpoint receptors that bind to their ligands CD80(B7-1) and CD86(B7-2), as well as programmed death ligands 1 and 2 (PD-L1, PD-L2), respectively, leading to tumor cell tolerance and the downregulation of effector T cells [19]. PD-1 is a protein receptor expressed by T cells, B cells, NK cells, DCs, and monocytes, and PD-L1 is overexpressed by tumor cells to decrease host immune response [20].

**Figure 1:**
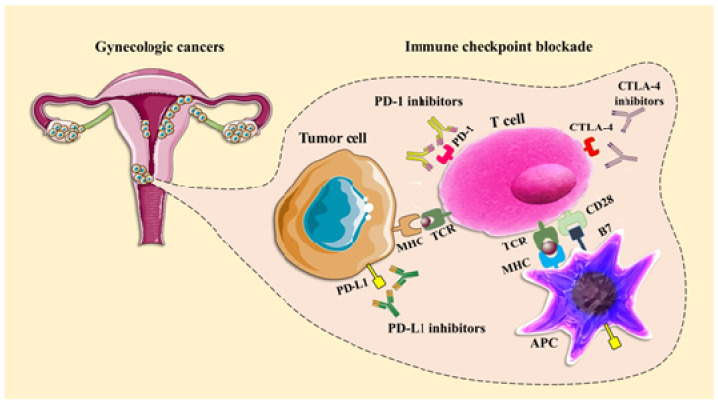
The mechanism of action of programmed cell death protein-1 (PD-1), programmed death ligand-1 (PD-L1), and cytotoxic T-lymphocyte-associated protein 4 (CTLA-4) inhibitors in cancer immunotherapy. Gynecologic cancers comprise a group of cancers that begin in the female reproductive system. The activation of T cells is mediated by the interaction of the T cell receptor (TCR) and the CD28 receptor with major histocompatibility complex (MHC) and the B7 co-stimulatory molecule located on the antigen-presenting cells (APCs). The interaction of CTLA-4 with the B7 molecule initiates an inhibitory signal, which is effectively blocked by CTLA-4 inhibitors. On the other hand, the negative regulation of T cells resulting from PD-1/PD-L1 interaction between T cells and tumor cells is suppressed by PD-1/PD-L1 inhibitors. CTLA-4 and PD-1/PD-L1 blocking antibodies have been shown to exert clinical antitumor activity in patients with gynecologic cancers. The figure is modified from Servier Medical Art (https://smart.servier.com).

Anti-PD-1 and -PD-L1 antibodies have a role in T cell activation or apoptosis and maintain tolerance in the peripheral immune system [21]. Cytotoxic T-lymphocyte-associated protein 4 and PD-1 antagonists contribute to the progress of effective antitumor immune responses when administered as monotherapy or supplementary therapy, depending on the type of tumor [22] (Figure 1).

## 3. Cervical Cancer

### 3.1. Risk Factors and Clinical Features

Cervical cancer (CC) is the eighth most common cancer on a worldwide scale of high incidence malignancies and is the most common gynecological cancer in developing countries [23]. The growing occurrence of the disease in developing countries is associated with several risk factors, including having numerous sexual partners, young age at first instance of intercourse, infrequent use of condoms, low socioeconomic status, smoking, and infection with human papillomavirus (HPV) [24]. Low-risk HPV types include types 6, 11, 42, 43, and 44. High-risk HPV types include types 16, 18, 31, 33, 34, 35, 39, 45, 51, 52, 56, 58, 59, 66, 68, and 70 [25,26]. Vaccines against HPV are essential to prevent the development of CC and other gynecologic cancers and to protect against HPV types 16 and 18. It has been estimated that vaccines may prevent 70% of CC, 60% of vaginal cancers, and 40% of vulvar cancers [27]. According to the current guidelines, pap smear and HPV tests are the most efficient options for CC screening [28]. The main clinical presentations in young patients with CC are abnormal—usually postcoital bleeding and an expanding cervical mass. The incidence of adenocarcinoma is currently on the rise [29]. Surgery is the main treatment for CC patients. Despite the potency of the agents used, chemotherapy alone is rarely curative and should be considered complementary only. Recently, a better understanding of the interactions between the tumor, the host’s immune system, and the development of ICIs have stimulated interest in the use of immunotherapy in CC [30].

### 3.2. Pre-Clinical Studies

The subtypes, levels, and locations of TILs may play several roles in CC development. Piersma et al. reported that lower numbers of CD8+ T TILs were associated with lymph nodes metastases in advanced CC patients [31]. Liang et al. described the same association. They enrolled a total of 137 patients with stage Ib2 and IIa2 CC in their study. Changes in TILs before and after neoadjuvant chemotherapy (NACT) and their prognostic importance in patients with advanced CC treated with NACT were evaluated. Foxp3+ T cell numbers significantly decreased in both intra-tumoral and peri-tumoral areas after NACT, while CD8+ T cell numbers remained stable [32]. Meng et al. investigated PD-L1, PD-1, and HPV expression by immunohistochemical (IHC) staining in CC and the normal cervix. They showed that CC tissues had more positive PD-L1, PD-1, and CD8 cells compared to healthy tissues, particularly those strongly stained for HPV. PD-L1, PD-1, and CD8 were found more frequently in advanced tumors, lymphoid nodes tumors, and vascular invasion [33]. Anggraeni et al. found a negative association between Fas ligand (FasL) expression and TIL levels in squamous cell carcinoma or adenocarcinoma tissues from patients with CC, which might indicate FasL-induced TIL apoptosis in tumor tissue [34]. The strong negative association between FasL and the presence of TILs sheds light on the interactions between tumor cells and their surroundings in CC. More recently, Li et al. examined 54 CC patients to determine whether the expression of the inhibitory receptor on the surface of CD8 TILs was associated with any clinical characteristics. A higher number of differentiated T cells (CD27, CC chemokine receptor7 (CCR7), and CD45RA) were found in bulk CD8 TILs that were related to high-grade CC [35].

### 3.3. PD-1 Inhibitors

Several clinical trials have investigated the antitumor activity of pembrolizumab and its safety in CC. In a phase 1 study, pembrolizumab showed antitumor activity, with a 17% objective response rate (ORR), and exhibited a safety profile, with 75% of patients experiencing immune-related adverse events (irAEs) [36]. A phase II trial assessed the efficacy and tolerability of nivolumab in cervical carcinoma patients [37]. Twenty-six patients with recurrent CC were enrolled to receive 3 mg/kg nivolumab every two weeks. Thirty-six percent of patients had stable disease (SD) with progression-free survival (PFS) and overall survival (OS) rates at six months of 16% and 78.4%, respectively. Nivolumab monotherapy showed significant improvement, with an appropriate safety profile in patients with recurrent CC.

Up to February 2020, several ongoing clinical trials have assessed the efficacy and possible adverse effects of nivolumab, pembrolizumab, and their combination with chemotherapy and radiotherapy compared to the standard treatments for cervical carcinoma. Selected ongoing trials are shown in Table 1. An interesting strategy is the combination of pembrolizumab with other types of treatments, such as chemotherapy and radiation therapy. Chemotherapeutic agents, antiangiogenic agents, and radiation can destroy tumor cells, release immune-stimulating tumor antigens, and especially produce an immunogenic response [38].

### 3.4. Combination Therapy of PD-1 Inhibitors with Chemotherapy and Radiotherapy

One interesting strategy is to combine PD-1 inhibitors with other types of treatments. Such an option is currently being evaluated in a phase II study in patients with advanced CC (ClinicalTrials.gov identifier: NCT02635360). Pembrolizumab will be administered concurrent with or subsequent to chemoradiation. Through the release of tumor antigens, tumor DNA, and cytokines into the tumor microenvironment, radiation augments the antitumor immune response to affect both the targeted lesion and distant sites of metastatic disease. Currently, combinations of chemotherapy and immunotherapy agents such as bevacizumab (anti-VEGF) or pembrolizumab for PD-1 positive cells are available options in CC patients. In addition to the crucial role in promoting the growth of tumor vessels, the vascular endothelial growth factor (VEGF) is also immunosuppressive. VEGF can inhibit the function of T cells, increase the recruitment of regulatory T cells (Tregs) and myeloid-derived suppressor cells (MDSCs), and hinder the differentiation and activation of dendritic cells (DCs) [38]. Another approach is to combine PD-1 inhibitors with radiation therapy. A phase I trial evaluated pembrolizumab as a monotherapy and in combination with hypofractionated radiation therapy (HFRT), or in combination with cyclophosphamide (CTX) or with CTX + HFRT in patients with advanced solid tumors, including CC. This study adopted a dose escalation design and no dose-limiting toxicity (DLT) was observed. The most common irAEs were fatigue (24.1%), arthralgia (12.1%), and nausea (10.3%). In total, 40.9% (*n* = 9) of the patients who received pembrolizumab + HFRT and 9.5% (*n* = 2) of the patients who received pembrolizumab monotherapy had a partial response (PR), suggesting that response to treatment was enhanced by the addition of HFRT [39]. An open-label phase II study named study of pembrolizumab, radiation and immune modulatory cocktail in cervical/uterine cancer (PRIMMO) is evaluating the combination of PD-1 blockade, radiation, and immunomodulation in patients with recurrent or refractory CC. The synergy between checkpoint blockade and radiation has the potential to expand the role of radiation in advanced and metastatic CC. Tumor regression outside of the irradiated field, known as the abscopal effect, is mediated by lymphocytes and enhanced by checkpoint blockade. Treatment consists of a daily intake of vitamin D, lansoprazole, aspirin, cyclophosphamide, and curcumin, starting 2 weeks before the first pembrolizumab dose. Pembrolizumab is administered 3-weekly for a total of 6 cycles. Radiation (3 × 8 Gy) is given on days 1, 3, and 5 of the first pembrolizumab dose. The primary endpoint is the ORR at week 26 and the secondary endpoints include safety, ORR at week 26, best overall response, PFS, OS, and quality of life. This ongoing trial will end in 2022 [40].

### 3.5. PD-L1 Inhibitors

Avelumab, atezolizumab, and durvalumab are the PD-L1 inhibitors tested in clinical trials in CC. In a phase I study, Rotman et al. are evaluating the safety, toxicity, and efficacy of low escalation durvalumab in CC. Three escalating dose levels of intratumorally (i.t.) injected durvalumab will be tested, i.e., 5, 10, and 20 mg (three patients per dose level, with an additional three at the highest tolerated dose). The primary endpoint of this ongoing phase I study is safety. Evidence of the safety and biological efficacy of durvalumab may expand adjuvant therapy options for cervical cancer patients [41].

### 3.6. Combination Therapy of PD-L1 Inhibitors with Chemotherapy and Radiotherapy

In a phase 1 trial, Mayadev et al. are investigating the efficacy of atezolizumab administered in combination with chemoradiation for node-positive locally advanced CC [42]. This trial has two experimental arms. Arm A will receive one dose of atezolizumab prior to chemotherapy with cisplatin and then two subsequent doses of atezolizumab, while arm B will receive three doses during chemotherapy. Patients will be monitored for two years to evaluate results. The study hypothesis is that there may be a difference in clonal expansions of TCR beta repertoires in the peripheral blood at day 21 between priming and concurrent atezolizumab and chemoradiation therapy in arm A vs. concurrent atezolizumab and chemoradiation therapy in arm B. In a phase II study, Friedman et al. assessed atezolizumab in combination with bevacizumab in patients with recurrent or metastatic CC [43]. Targeting VEGF via bevacizumab in combination with PD-L1 blockade may improve clinical outcomes by enhancing T cell infiltration into tumors; this has been demonstrated in patients with recurrent CC. A total of 11 patients were recruited and treated with bevacizumab (15 mg/kg every three weeks) and atezolizumab (1200 mg/kg every three weeks). Median PFS (mPFS) and median OS (mOS) were 2.9 months and 9 months, respectively. The clinical benefit with the use of atezolizumab was modest. The first-line standard treatment for patients with metastatic or recurrent CC is chemotherapy with cisplatin or paclitaxel plus bevacizumab, with a short median OS (16.8 months) and PFS (8.2 months). The addition of atezolizumab to these chemotherapeutic agents may improve OS rates. An ongoing phase III study by Grau et al. is evaluating the efficacy of chemotherapy plus atezolizumab (platinum plus paclitaxel with bevacizumab and atezolizumab) versus chemotherapy alone (platinum plus paclitaxel and bevacizumab) in metastatic or recurrent CC [44].

Another interesting strategy in the treatment of CC patients is to combine an anti-PD-L1 antibody, such as durvalumab, with tremelimumab (an anti-CTLA-4 antibody) (ClinicalTrials.gov identifier: NCT01975831).

Currently, several ongoing trials are investigating the efficacy and safety of atezolizumab in combination with radiotherapy and chemotherapy in CC; some of them are presented in Table 2. The aim of NCT03614949 is to determine whether treatment with atezolizumab and radiation therapy could improve ORR compared to atezolizumab alone in patients with recurrent or metastatic CC [45]. Recently, two ongoing clinical trials are investigating the safety and efficacy of avelumab in CC. The lytic activation to enhance neoantigen-directed therapy (LATENT) study aims to assess the efficacy of avelumab with valproic acid (VPA) in virus-associated cancers, including cervix cancer [46]. VPA is a histone deacetylase (HDAC) inhibitor that has an anticancer effect. Previous in vitro studies showed that VPA prevents the growth of CC cells through caspase-dependent apoptosis and inhibition of growth [47]. A total of 39 patients will participate in this study and will receive VPA (12.5 mg/kg) and avelumab (10 mg/kg) for 2 years. The ALARICE trial will evaluate the efficacy of avelumab with axitinib in recurrent CC. A total of 23 participants will receive avelumab (10 mg/kg) every two weeks and axitinib (5 mg/kg) in up to 12 cycles (NCT03826589). These clinical trials will help determine the optimal dose and select the target population for subsequent studies (Table 2).

## 4. Ovarian Cancer

### 4.1. Risk Factors and Clinical Features

Ovarian cancer (OC) is the seventh most common cancer among women, with OS rates under 45% [48]. Nowadays, there are no verified preventative measures and no beneficial screening tools [49]. Primary therapies for OC include surgery with or without NACT to eradicate as many cancer cells as possible [50]. In recurrent OC, chemotherapy, antiangiogenic agents, and poly (ADP-ribose) polymerase inhibitors are used, and immunotherapies are currently being tested [51]. The available immunotherapy options against OC are cancer vaccines, adoptive cell therapy, and ICIs [52].

### 4.2. Pre-Clinical Studies

TILs express the negative regulatory receptor, PD-1, which is upregulated upon T cell activation and inhibits T effector functions, while cancer cells express its ligand PD-L1. Cancer tissue expression of PD-L1 is correlated with reduced intraepithelial TILs and low OS in OC [53]. Higher levels of TILs in OC are a predictive marker of improved OS, whereas increases in Treg cells are associated with poor outcomes [54]. CD4+ and CD8+ TILs have long been known to exist in OC. Zhang et al. examined 186 specimens from advanced OCs and observed that the 5-year survival rate in the 55% of patients whose tumors contained CD3^+^ TILs was 38% and was 4.5% in patients whose tumors contained no TILs [55]. In another study on 70 specimens of OC, higher levels of PD-L1 were associated with prognosis and the presence of CD8+ TILs was negatively correlated with PD-L1 expression. In this study, TILs were isolated from OC tumors and showed higher PD-1 expression, with impaired production of TNF-α and IFN-γ [56]. Wang et al. studied PD-L1 expression and CD3+, CD4+, and CD8+ TIL infiltration in 107 advanced OC patients via IHC analysis. They demonstrated that a higher number of intraepithelial TILs was a prognostic factor for longer OS, while PD-L1 expression was associated with a shorter OS [57]. High-grade serous ovarian cancers (HGSOCs) with BRCA1/2 mutation exhibited a higher mutation burden and may harbor several tumor-specific neoantigens [58]. Immunohistochemistry examinations showed that BRCA1/2-mutated cancers have considerably higher numbers of CD3+ and CD8+ TILs and higher levels of PD-L1 and PD-1 expression in TILs compared to homologous recombination-proficient tumors [59].

### 4.3. PD-1 Inhibitors

In a phase II trial, Hamanishi et al. assessed the anticancer activity of nivolumab in OC [60]. Nivolumab was administered to 20 platinum-resistant patients at a dose of 1 or 3 mg/kg every two weeks for up to six cycles. OS and PFS were 20.0 and 3.5 months, respectively. The KEYNOTE-028 trial evaluated the tolerability and effectiveness of pembrolizumab monotherapy in advanced OC [61]. The results showed that ORR was 11.5%, mPFS was 1.9 months, and mOS 13.8 months. In a phase 1 trial, Liu et al. examined the clinical efficacy, safety, and tolerability of atezolizumab in recurrent ovarian and uterine cancers [62]. Atezolizumab proved to be well-tolerated in patients with recurrent OC and it may have a clinical activity that calls for further investigation. Currently, several ongoing trials are assessing the safety and efficacy of nivolumab in OC; some of them are shown in Table 3.

### 4.4. Monotherapy or Combination Therapy with PD-L1 Inhibitors in Ovarian Cancer

In a phase I study, Zimmer et al. evaluated the efficacy of durvalumab in combination with a PARP inhibitor, olaparib, and a VEGFR1–3 inhibitor, cediranib, in recurrent OC. The authors hypothesized that enhanced DNA damage caused by olaparib and reduced VEGF signaling caused by cediranib would complement the antitumor activity of durvalumab, and that the 3-drug combination would be tolerable. Cediranib was taken intermittently (5 days on/2 days off) at 15 or 20 mg with durvalumab (1500 mg IV every 4 weeks) and olaparib tablets (300 mg twice daily). The primary endpoint was the recommended phase 2 dose (RP2D) and the secondary endpoints were ORR, pharmacokinetic (PK), and correlative analyses. In total, 7 (of 9) OC patients were treated and no patients experienced dose-limiting toxicities. Common irAEs were hypertension, anemia, and lymphopenia. Four patients had PRs (44%) and 3 had SD. No significant effects of the co-administration with olaparib or cediranib or from the presence of durvalumab were identified. [63]. Inhibition of histone deacetylase 6 (HDAC6) suppresses the growth of AT-rich interaction domain 1 (ARID1A) (a mutated epigenetic regulator) in tumors and modulates the tumor immune microenvironment. Fukumoto et al. showed that inhibition of HDAC6 synergizes with PD-L1 inhibitors in ARID1A-inactivated OC. These findings suggest a rationale for combining epigenetic modulators and PD-L1 inhibitors against OC [64].

Currently, several clinical trials are assessing the clinical activity and safety of PD-L1 inhibitor monotherapy or in combination with chemotherapeutic agents such as bevacizumab, paclitaxel or carboplatin, cobimetinib (the mitogen-activated protein kinase enzymes inhibitor) [65], and platinum-based chemotherapy in OC (Table 4) [66,67].

The KGOG 3045 study aims to investigate durvalumab therapy in platinum-resistant recurrent OC patients [65,68]. In total, 68 patients have been randomized into four groups: group 1 will receive olaparib and cediranib, group 2 will receive olaparib and durvalumab, group 3 will receive durvalumab plus chemotherapy, and group 4 will receive durvalumab and tremelimumab plus chemotherapy. The primary endpoint is to measure the objective response rate, while secondary endpoints are PFS, OS, immune-related response criteria, and duration of response. The DUO-O trial aims to assess durvalumab therapy in combination with bevacizumab and chemotherapy in OC patients [69]. The KGOG3046 study is being conducted to evaluate durvalumab plus chemotherapy and tremelimumab in advanced OC [70]. This phase 2 trial involves 24 participants and will end in May 2021.

### 4.5. Combination Therapy of PD-1 Inhibitors with Chemotherapy and Radiotherapy

To date, single agent anti-PD-1/PD-L1 therapy has shown limited activity in recurrent epithelial OC versus combination with chemotherapy and radiotherapy. Combination strategies of PD-1/PD-L1 inhibition with antiangiogenic therapy have the potential for synergistic activity through modulation of the microenvironment and represent a potential therapeutic opportunity in this disease. Currently, ongoing trials are evaluating the combination of nivolumab with chemotherapeutic agents (bevacizumab, carboplatin or paclitaxel), immunotherapy tools (Wilms’ tumor gene 1 (WT1) and NeoVax vaccines), and poly (ADP-ribose) polymerase inhibitors such as rucaparib in OC [71,72]. Liu et al. enrolled a total of 38 patients to evaluate the activity of combination therapy with nivolumab and bevacizumab in women with relapsed OC. Patients received intravenous nivolumab and intravenous bevacizumab once every two weeks. The primary endpoint was ORR and secondary endpoints were ORR, PFS, and safety. Of the 38 women enrolled, 18 had platinum-resistant and 20 patients had platinum-sensitive disease, while 11 patients experienced a confirmed response to nivolumab with bevacizumab (ORR, 28.9%). The ORR was 40.0% (19.1–64.0%) in platinum-sensitive and 16.7% in platinum-resistant participants. Median PFS was 8.1 months. The nivolumab with bevacizumab combination appeared to show activity in patients with relapsed OC [73].

Some studies combined PD-1 inhibitors with CTLA-4 inhibitors such as ipilimumab to improve efficacy. Zamarin et al. evaluated ipilimumab plus nivolumab combination therapy and compared it with nivolumab monotherapy in women with persistent or recurrent OC. A total of 100 patients received either intravenous nivolumab (every two weeks) (*n* = 49) or nivolumab plus ipilimumab for four doses (every three weeks) (*n* = 51) followed by fortnightly maintenance with nivolumab for a maximum of 42 doses. The median PFS was 2 months in the nivolumab group and 3.9 months in the nivolumab plus ipilimumab groups. Compared with nivolumab monotherapy, the combination of nivolumab and ipilimumab in OC resulted in a better response rate and longer PFS [74].

Cemiplimab, another PD-1 inhibitor, is being tested in combination with REGN4018 (a human bispecific antibody targeted against mucin16, an antigen expressed in OC and several other tumors, and CD3 on T cells) to evaluate its efficacy and safety in OC.

The NCT03564340 trial will assess the administration of REGN4018 alone or in combination with cemiplimab in OC platinum-resistant patients. A total of 264 patients will participate in this phase 1/2 study. The primary endpoint of this trial is to evaluate the preliminary efficacy of monotherapy with REGN4018 and the combination of REGN4018 with cemiplimab; the secondary endpoint will include will be to estimate response length, disease control rate, and PFS (Table 3) [66].

## 5. Uterine Cancer

### 5.1. Risk Factors and Clinical Features

Uterine cancer (UC) is the fourth most common cancer in women in the USA. In 2019, approximately 61,880 new cases of UC and 12,160 deaths from the disease were estimated [75], with the number of UC cases expected to increase in older populations. UC includes endometrial adenocarcinoma (EA) (the most common type), adenosquamous carcinoma, papillary serous carcinoma, and uterine sarcoma [76,77]. Patients in stage I disease have a good prognosis and can be cured with surgery therapy. Patients in later stages (stage III or IV) have a poor prognosis, with five-year OS rates ranging from 47 to 69% (stage III) and 15 to 17% (stage IV) [78]. An unbalanced increase in estrogen levels due to early menstruation, delayed menopause, obesity, and exposure to tamoxifen is the most critical risk factor for UC [79]. Surgery is often the main treatment in most patients [80]. Comorbidities such as diabetes and obesity are considered as risk factors for the progression of UC and are essential regarding the choice of surgery to ensure a positive outcome [81]. Traditionally, UC treatment was managed by grade and histology. Recent studies showed that cancers of the same stage and histology have very distinct molecular and genomic profiles. Recently, investigations into subgroups of UC, including polymerase epsilon (POLE)-ultramutated and microsatellite instability-hypermutated (MSI-H) cancers, revealed that combinations of ICIs and chemotherapy in association with small-molecule tyrosine kinase inhibitors (TKIs) lead to strong antitumor immune responses [82].

Existing data suggest that UC is sufficiently immunogenic to be a logical candidate for immunotherapy. Anticancer vaccines, adoptive cell transfer, bispecific T cell engager antibodies, and ICIs are the most critical immunotherapy methods in UC therapy [83]. UC cells can stimulate immune checkpoints to evade a host’s immune response, activating negative feedback mechanisms and creating an immunosuppressed environment [84].

### 5.2. Pre-Clinical Studies

PD-1 and PD-L1 are overexpressed in 75% and 25–100% of UC patients, respectively [85]. PD-1/PD-L1 targeting has garnered enthusiasm as an approach that improves the antitumor immune response. Reddy et al. examined the expression of PD-L1 in human uterine tumors. PD-L1 IHC staining was performed on a tissue microarray of 101 normal and malignant uterine tissue samples. PD-L1 was positive in 34.4% of endometrial adenocarcinomas and in 37.8% of squamous cell carcinomas [9,84]. Another study by Shanes et al. evaluated 49 uterine smooth-muscle tumors for PD-L1 expression and TIL infiltration. In total, 70% of leiomyosarcomas and 14% of atypical leiomyomas demonstrated PD-L1 expression [86]. Engerud et al. reported PD-L1 and PD-1 expression in 59% and 63% in primary tumors, respectively, in a cohort of 700 patients. However, neither had any impact on survival in microsatellite stable (MSS) and MSI tumors [87]. Mo et al. assessed PD-1, PD-L1, and PD-L2 expression in 35 healthy endometrial tissues and 75 EA tissues. PD-1 was not expressed in the tumor or in the healthy endometrial tissues. Moreover, 14.3% of healthy endometrial tissues and 17.3% of EA tissues were positive for PD-L1 expression, while 20.0% of healthy endometrial tissues and 37.3% of EA tissues were positive for PD-L2 expression [88]. Sungu et al. found positive staining for PD-L1 (36%), PD-L2 (64.4%), and PD-1 expression (61.6%) in EA cells, and positive PD-L1 (36.2%) and PD-L2 (93.2%) expression in immune cells [89]. Collectively, the findings show that the synergism between PD-1, PD-L1, and PD-L2 could be a possible target for immunotherapy in advanced EA.

### 5.3. PD-1 Inhibitors

Pembrolizumab could be beneficial for 20–30% of patients with advanced UC [90]. The reasons for this lie in the immunosuppressive effects exerted by UC tumors on the microenvironment and an altered tumor vasculature. The KEYNOTE-028 study was the first published trial designed to assess the efficacy of pembrolizumab in patients with advanced UC [91]. A total of 477 patients were treated with pembrolizumab (10 mg/kg) every two weeks for two years. The ORR was 13%. Three patients achieved a PR and three patients had SD. The 6-month PFS and 6-months OS rates were 19.0% and 68.8%, respectively. Adverse events were observed in 54.2% of patients. The most common side effects were pruritus, asthenia, and fatigue.

### 5.4. Combination Therapy with PD-1 Inhibitors

There is established evidence that the tumor microenvironment (TME) plays a crucial role in modulating an antitumor immune response, making a compelling case for combinatorial approaches to improve responses to ICIs [92]. Recent pre-clinical and clinical data show that the combination of radiotherapy and chemotherapy with ICIs is associated with acceptable toxicity, and that these agents could improve the effect of ICIs, mainly when administered concomitantly. Co-inhibition of VEGF via lenvatinib and of PD-1 signaling via pembrolizumab could be an effective antitumor strategy. In a mouse model, lenvatinib significantly decreased the population of tumor-associated macrophages (TAMs) and increased the percentages of CD8-positive T cells, which led to enhanced antitumor activity by PD-1 inhibitors [93]. The combination of pembrolizumab with axitinib, lenvatinib, and paclitaxel was evaluated in metastatic UC [94]. In a phase 1/2 trial, Makker et al. investigated the anti-UC activity of pembrolizumab. A total of 23 patients received pembrolizumab (200 mg) every three weeks combined with lenvatinib (20 mg per day). The ORR was 48%; hypertension, fatigue, arthralgia, diarrhea, and nausea were the most common side effects [95]. Another study is underway to determine the efficacy and safety of pembrolizumab in combination with axitinib in recurrent UC. In this phase II study, 26 patients will be treated with pembrolizumab (200 mg) and 5 mg axitinib. The primary endpoint is ORR at week 12 and the secondary endpoints are OS and average PFS [96]. The KEYNOTE-775 study is assessing pembrolizumab in combination with lenvatinib vs. placebo in patients with UC. In this phase 3 study, 780 patients will receive pembrolizumab (200 mg) plus lenvatinib (20 mg) during each 21-day cycle. The primary endpoints are PFS and OS, while secondary endpoints are ORR, health-related quality of life (HRQoL) score, and irAEs [97].

In the phase 2 PRIMMO Study, Tuyaerts et al. are evaluating the antitumor efficacy of pembrolizumab in combination with radiotherapy and immunomodulation in UC patients [40]. Patients will receive pembrolizumab over 3 weeks for a total of 6 cycles, with radiation given on days 1, 3, and 5. The primary endpoint is the ORR at week 26. The secondary endpoints are safety, overall response, and PFS. The aim of the phase 1 PAM study is to evaluate the clinical efficacy of pembrolizumab in UC patients [98]. Twenty patients (ten patients with deficient mismatch repair and ten patients with polymerase ε mutation) will receive 200 mg/kg pembrolizumab for a total of two administrations per patient. The primary endpoint is the response rate of the tumor and the secondary endpoint is the ORR of the tumor, as determined by MRI.

### 5.5. Ongoing Trials of Combination Therapy with PD-L1 Inhibitors

The endometrial bevacizumab, atezolizumab, rucaparib (EndoBARR) trial is under way to demonstrate the clinical activity of the combination of atezolizumab with rucaparib and bevacizumab in uterine sarcoma. In this phase 2 single-group study, 30 patients will receive 1200 mg atezolizumab for 21 days, and on day one will receive 15 mg/kg bevacizumab and 600 mg rucaparib twice daily by continuous dosing. The primary outcome will measure ORR and the secondary outcomes will measure PFS and OS [99]. In a phase I study, Fleming et al. assessed the efficacy of atezolizumab in 15 UC patients. Patients received atezolizumab (15 mg, every three weeks). Two patients achieved PR, while two others had SD, with an ORR of 13%. The durations of response were 7.3 and 8.1 months, respectively. The mPFS was 1.7 months and mOS was 9.6 months. Only two patients had irAEs (colitis and rash). No grade 4–5-related irAE occurred [100]. Table 5 summarizes the ongoing trials (up to February 2020) of PD-L1 inhibitors in UC and their associations with chemotherapy, radiotherapy, TKIs, and mTOR inhibitors.

## 6. ICIs and Drug Resistance

Failure of ICI therapy can result from (1) inadequate generation of TILs, (2) weak specific T cell function, and (3) reduction in T cell memory formation [45]. Primary drug resistance and acquired drug resistance are crucial issues in ICI treatment. Drug resistance in tumor cells could occur through several mechanisms, including disabling mutations in Janus kinase 1 (JAK1), JAK2, and beta 2-microglobulin (B2M) genes; PD-L1 upregulation; decreased major histocompatibility complex (MHC) expression; increased PD-L2 levels on PD-L1 negative cancer cells; stromal remodeling; epithelial–mesenchymal transition (EMT); and host cells (including T cells) expressing PD-L1 [101]. PD-L1 expression may be triggered in tumor cells through several signaling pathways, such as ALK/STAT3, PI3K/AKT, and MEK/ERK/STAT1. Recently, Hugo et al. described primary PD-1 resistance mechanisms associated with a set of immune-suppressive cytokines, EMT transcription factors, and pro-angiogenic factors [102]. Frenel et al. reported that pembrolizumab treatment in 24 CC patients with PD-L1-positive tumors resulted in an ORR of only 17% [103].

The transforming growth factor beta (TGF-β) pathway is another mechanism that inhibits CD4+ T cells and cytotoxic T cells, while promoting the development, production, and activity of Treg cells. TGF-β negatively affects adaptive immunity by specifically inhibiting CD8+ T cells and CD4+ T cells from clonal expansion and cytotoxicity. Pre-clinical studies found that low ORRs to PD-L1 therapy were correlated with TGF-β signaling in fibroblasts in the metastatic stage of UC. Pan et al. found five possible biomarkers (CD48, SEPT1, ACAP1, PPP1R16B, and IL16) that are significantly associated with EMT and suggested that their high expression may decrease the response rate to ICIs in bladder cancer [104]. According to Gargiulo et al., the two subgroups of EC, the POLE ultramutated and MSI-H groups, have a higher number of neoantigens and TILs, presenting an enhanced immune microenvironment and a high mutation burden, meaning they could have a better rate of response to ICIs. They also observed that a smaller number of neoantigens were associated with different gene alterations, catenin (cadherin-associated protein), beta 1 (CTNNB1) and phosphatidylinositol-4,5-bisphosphate 3-kinase catalytic subunit alpha (PIK3CA) mutations, and MYC amplification, which are indicators of a lower ORR to ICIs [105].

Whereas cytotoxic T cells are known to present significant antitumor effects during checkpoint inhibition, some cancers with low MHC expression are responsive to therapy, suggesting that NK cells may also play a role. Immune checkpoints in NK cells demonstrate various expression patterns: (1) some are stably expressed; (2) some are generally absent or poorly expressed and are upregulated upon stimulation; (3) some are expressed normally and are further upregulated in particular contexts. Tumors usually escape T cell immune surveillance by downregulating the expression of MHC class I to compromise the tumor antigen presentation pathway, making these tumors difficult to recognize by T cells. MHC-I-null tumor cells are not attacked by T cells, but they are still targeting of NK cells. Recently, the physiological functions in tumor surveillance of NK cells, as well as the therapeutic potential of many NK cell surface receptors, have been illustrated (e.g., KIR, TIGIT, NKG2A, CD96, and PD-1), while those of many others remain to be shown (e.g., LAG-3 and TIM-3) [106]. Killer cell immunoglobulin-like receptors (KIRs) were the first NK cell immune checkpoints identified in 1990. Trials using anti-KIR or anti-NKG2A MAbs blocked the inhibitory signals generated by these receptors and restored the antitumor NK cell activity. Thus, the combined blockade of different checkpoints on T cells and NK cells may simultaneously activate both natural and acquired immunity [107]. Studies using flow cytometry, quantitative reverse-transcriptase PCR (qRT-PCR), and RNA-Seq for PD-1 expression evaluated NK cells in primary human tumor samples and demonstrated under various conditions that human and mouse NK cells consistently lack PD-1 expression, despite the significant upregulation of other regulatory markers, such as TIGIT [108]. Zhang et al. reported that inhibition of TIGIT, which is expressed by both T cells and NK cells, could promote the antitumor immunity of both T and NK cells [109]. The CD94/NK group 2 member A (NKG2A) heterodimeric receptor is one of the most important NK inhibitory receptors. NKG2A binds to HLA-E (a nonclassical HLA class I molecule), which presents peptides to other HLA class I molecules, such as HLA-G. Studies showed that NKG2A downregulation evades the HLA-E cancer immune checkpoint and increases the antitumor activity of NK cell infusions [110]. In addition, inhibition of PD-1 and PD-L1 has been shown to elicit a strong NK cell response that is necessary for the full therapeutic outcome of immunotherapy. The in vivo expression of PD-1 and PD-L1 on NK cells in cancer mouse models resulted in reduced NK cell responses and the formation of more aggressive tumors [111]. Hence, a growing body of evidence suggests that targeting NK cells in vivo is achievable and may provide an alternative or complementary immunotherapy approach to the ICIs.

Collectively, potential approaches for further developing highly reliable predictive biomarkers should facilitate patient selection and decision-making related to immune checkpoint inhibitor-based therapies. Although several investigations on predictive biomarkers have been designed and many are under way, clinical validation of recognized biomarkers is necessary.

## 7. Conclusions

Immune checkpoint inhibitors especially PD-1/PD-L1 inhibitors have shown promising antitumor activity in clinical trials as a treatment for gynecologic cancers. Several trials are testing their efficacy and possible irAEs in combination with chemotherapy and radiation therapy. The employment of ICIs in patients with gynecological malignancies is based on the IHC assays showing high expressions of PD-L1 and PD-1 in ovarian cancer [112,113]. The safety and efficacy of available PD1/PD-L1 inhibitors, including nivolumab, pembrolizumab, atezolizumab, avelumab, and durvalumab, are currently being assessed in several trials. While PD-1/PD-L1 inhibition offers a promising treatment option, there are several considerations for the use of checkpoint inhibitors. It is necessary to know the different toxicities associated with ICIs, because some of these side effects are serious and life-threatening. A new research area is determining the association between the rates of adverse effects with the response to treatment. Most responses occur within the early days of beginning treatment. Despite the impact of ICIs on cancer therapy, a low rate of response to these agents is observed in several malignancies, such as gastrointestinal cancers [114], breast cancer [115], and parts of genitourinary cancers, as we discussed earlier. The first approach assessed to increase the response rate to ICIs is the use of predictive or prognostic biomarkers, such as mutational burden, PD-L1 expression, and clinical characteristics. Another approach is the use of ICIs in combination with other therapeutics, such as radiotherapy, microbiota modifiers, antiangiogenic therapeutics, drugs targeting co-inhibitory receptors, oncolytic virotherapy, and small molecules, in order to achieve an increase of antitumor activity through the alteration of the tumor immune microenvironment [116]. As the results are encouraging, more clinical trials should be designed, contributing to the improvement of effective treatments for gynecological cancer patients.

## Figures and Tables

**Table 1:** Selected ongoing (up to February 2020) trials of nivolumab and pembrolizumab in cervical cancer.

Estimated Completion date	Title	Phase	Country	Indication	Endpoints	Clinical Trials. Gov. Identifier
2023	Combination of GX-188E Vaccination and Pembrolizumab in Patients with HPV 16 and/or 18+ Cervical Cancer	Phase 1/2	Republic of Korea	Advanced, inoperable, or metastatic cervical cancer	ORR, DLT evaluation for safety and tolerability PFS	NCT03444376
2025	Combination Pembrolizumab, Chemotherapy, and Bevacizumab in Patients with Cervical Cancer	Phase 2	United States	Recurrent, persistent, or metastatic (primary stage IVB) cervical cancer	ORR, PFS, OS	NCT03367871
2021	Pembrolizumab and Chemoradiation Treatment for Advanced Cervical Cancer	Phase 2	United States	Advanced cervical cancer	Change in immunologic markers, PFS, OS	NCT02635360
2023	Carboplatin-Paclitaxel-Pembrolizumab in Neoadjuvant Treatment of Locally Advanced Cervical Cancer (MITO CERV 3)	Phase 2	Italy	Locally advanced cervical cancer	2-year PFS, OS, clinical response rate, adverse events	NCT04238988
2022	Efficacy and Safety Study of First-line Treatment with Pembrolizumab (MK-3475) plus Chemotherapy Versus Placebo Plus Chemotherapy in Women with Persistent, Recurrent, or Metastatic Cervical Cancer (MK-3475-826/KEYNOTE-826)	Phase 3	United States	Persistent, recurrent, or metastatic cervical cancer	PFS, OS, ORR, DOR	NCT03635567
2022	Cabozantinib plus Pembrolizumab for Recurrent, Persistent, and/or Metastatic Cervical Cancer	Phase 2	United States	Recurrent, persistent, or cervical cancer	PFS, ORR, OS, incidence of emergent adverse events	NCT04230954
2022	Nivolumab in Association with radiotherapy and Cisplatin in Locally Advanced Cervical Cancers Followed by Adjuvant Nivolumab for up to 6 Months (NiCOL)	Phase 1/2	France	Locally advanced cervical cancer	ORR, PFS, disease-free survival (DFS)	NCT03298893
2023	BrUOG 355: Nivolumab to Tailored Radiation Therapy with Concomitant Cisplatin in the Treatment of Patients with Cervical Cancer	Phase 2	United States	Advanced cervical cancer	Feasibility of the incorporation of nivolumab with weekly cisplatin	NCT03527264
2019	Nivolumab in Treating Patients with Persistent, Recurrent, or Metastatic Cervical Cancer	Phase 2	United States	Stage IV, stage IVA, and stage IVB cervical cancer	Frequency of objective tumor response, incidence of adverse events	NCT02257528

Abbreviations: ORR; overall response rate, PFS; progression-free survival, DFS; disease-free survival, OS; overall survival.

**Table 2:** Ongoing trials (up to February 2020) of atezolizumab alone or in combination with chemotherapy in cervical cancer.

Estimated Completion Date	Title	Phase	Country	Indication	Clinical Trials. Gov. Identifier
July 2020	Doxorubicin Alone Versus Atezolizumab Alone Versus Doxorubicin and Atezolizumab in Recurrent Cervical Cancer	Phase 2	Belgium	Recurrent Cervical Cancer	NCT03340376
July 2022	Trial Assessing the Inhibitor of Programmed Cell Death Ligand 1 (PD-L1) Immune Checkpoint Atezolizumab (ATEZOLACC)	Phase 2	France	Locally Advanced Cervical Cancer	NCT03612791
December 2023	Platinum Chemotherapy Plus Paclitaxel with Bevacizumab and Atezolizumab in Metastatic Carcinoma of the Cervix	Phase 3	Finland	Metastatic Cervical Cancer	NCT03556839
August 2020	Atezolizumab and Bevacizumab in Treating Patients with Recurrent, Persistent, or Metastatic Cervical Cancer	Phase 2	United States	Stage IV-IVA-IVB Cervical Cancer	NCT02921269
November 2021	Atezolizumab Before and/or With Chemoradiotherapy in Immune System Activation in Patients with Node-Positive Stage IB2, II, IIIB, or IVA Cervical Cancer	Phase 1	United States	Different Stage of Cervical Cancer	NCT03738228

**Table 3:** Ongoing trials (up to February 2020) of nivolumab in ovarian cancer.

Estimated Completion Date	Title	Phase	Country	Indication	Clinical Trials. Gov. Identifier
2026	NeoVax with Nivolumab in Patients with Ovarian Cancer	Phase 1	United States	Primary peritoneal or fallopian tube ovarian cancer	NCT04024878
2021	A Study of WT1 Vaccine and Nivolumab For Recurrent Ovarian Cancer	Phase 1	United States	Recurrent ovarian Cancer	NCT02737787
2021	Tolerance of Intraperitoneal (IP) Nivolumab after Extensive Debulking Surgery and Hyperthermic Intraperitoneal Chemotherapy (HIPEC) in Patients with Advanced Ovarian Carcinoma (ICONIC)	Phase ½	France	Advanced ovarian cancer	NCT03959761
2030	A Study in Ovarian Cancer Patients Evaluating Rucaparib and Nivolumab as Maintenance Treatment Following Response to Front-Line Platinum-Based Chemotherapy (ATHENA)	Phase 3	United States	Maintenance treatment for ovarian cancer	NCT03522246
2020	Nivolumab with or without Ipilimumab in Treating Patients with Persistent or Recurrent Epithelial Ovarian, Primary Peritoneal, or Fallopian Tube Cancer	Phase 2	United States	Recurrent Ovarian carcinoma	NCT02498600

**Table 4:** Ongoing trials (up to February 2020) of PD-L1 inhibitor monotherapy or in combination with chemotherapy in ovarian cancer.

Estimated Completion Date	Title	Phase	Country	Indication	Clinical Trials Gov. Identifier
2021	Avelumab and Talazoparib in Untreated Advanced Ovarian Cancer (JAVELIN OVARIAN PARP 100)	Phase 3	United States	Advanced Ovarian Cancer	NCT03642132
2019	A Study of Avelumab Alone or in Combination with Pegylated Liposomal Doxorubicin versus Pegylated Liposomal Doxorubicin Alone in Patients with Platinum Resistant/Refractory Ovarian Cancer (JAVELIN Ovarian 200)	Phase 3	United States	Resistant/Refractory Ovarian Cancer	NCT02580058
2023	A Trial of Hu5F9-G4 with Avelumab in Ovarian Cancer	Phase 1	United States	Advanced Solid-Tumor Ovarian Cancer	NCT03558139
2021	Phase 1b/2 Study of Avelumab with or without Entinostat in Patients with Advanced Epithelial Ovarian Cancer	Phase 1/2	United States	Epithelial Ovarian Cancer Peritoneal Cancer Fallopian Tube Cancer	NCT02915523
2022	Atezolizumab with Neoadjuvant Chemotherapy for Patients with Newly Diagnosed Advanced-Stage Ovarian Cancer (AdORN)	Phase 1/2	United States	Advanced-Stage Ovarian Cancer	NCT03394885
2022	Atezolizumab with Bevacizumab and Chemotherapy vs. Bevacizumab and Chemotherapy in Early Relapse Ovarian Cancer	Phase 3	Germany	Recurrent Ovarian Carcinoma	NCT03353831
2022	A Clinical Study of Cobimetinib Administered in Combination with Niraparib, with or without Atezolizumab, to Patients with Advanced Platinum-Sensitive Ovarian Cancer	Phase 1	United States	Advanced Platinum-sensitive Ovarian Cancer	NCT03695380

**Table 5:** Ongoing trials (up to February 2020) of PD-L1 inhibitors in uterine cancer.

Treatment Setting	Phase	Estimated Completion Date	Endpoints	Clinical Trials. Gov. Identifier	Enrollment
Avelumab Talazoparib	2	2024	PFS OS irAEs	NCT02912572	70 participants
Carboplatin Paclitaxel Avelumab	2	2023	PFS OS Number of patients with complete and PR	NCT03503786	120 participants
PARP Inhibitor and Durvalumab	2	2023	PFS ORR OS irAEs	NCT03951415	55 participants
Durvalumab Tremelimumab	2	2021	ORR	NCT03015129	80 participants
Bevacizumab Atezolizumab	2	2023	Number of patients with complete and PR PFS OS	NCT03526432	55 participants
Carboplatin, Cyclophosphamide, Atezolizumab	1	2020	Toxicity ORR	NCT02914470	12 participants
Rucaparib, Bevacizumab, Atezolizumab	2	2026	ORR PFS irAEs OS	NCT03694262	30 participants

Abbreviations: ORR; overall response rate, PFS; progression-free survival, DFS; disease-free survival, OS; overall survival, irAEs; immune-related adverse events, PR; partial response.

## Abbreviations

FDA	the US food and drug administration
TILs	tumor-infiltrating lymphocytes
T-reg	regulatory T cells
TAM	tumor-associated macrophages
CTLA-4	cytotoxic T-lymphocyte antigen 4
PD-1	programmed cell death protein 1
PD-L1	Programmed death-ligand
NACT	neoadjuvant chemotherapy
SD	stable disease
PFS	progression-free survival
OS	overall survival
irAEs	immune-related adverse events
mPFS	median PFS
mOS	median OS
ORR	overall response rate
HPV	human papillomavirus
ICIs	immune checkpoint inhibitors
PR	partial response
ORR	overall response rate
VPA	valproic acid
OC	ovarian cancer
UC	uterine cancer
EC	endometrial cancer
CTNNB1	catenin beta 1
PIK3CA	phosphatidylinositol-4,5-bisphosphate 3-kinase catalytic subunit alpha
HGSOCs	high grade serous ovarian cancers
DOR	response length
ACT	adoptive cell transfer
BiTE	bispecific T cell engager
UC	uterine cancer
EC	endometrial cancer
MSI-H	the microsatellite instability-hyper-mutated
POLE	the polymerase epsilon
TKIs	tyrosine kinase inhibitors
MSS	microsatellite stable
HRQoL	health-related quality of life
pCR	pathological complete response
ADCC	antibody-dependent cell-mediated cytotoxicity
B2M	beta-2 microglobulin
EMT	epithelial-mesenchymal transition
IPRES	primary PD-1 resistance
HFRT	hypofractionated radiation therapy
CTX	cyclophosphamide
DLT	dose-limiting toxicity
PK	pharmacokinetic
